# Crystal structure of 1-[(6-chloro­pyridin-3-yl)sulfon­yl]-1,2,3,4-tetra­hydro­quinoline

**DOI:** 10.1107/S2056989015008099

**Published:** 2015-05-23

**Authors:** S. Jeyaseelan, H. R. Rajegowda, R. Britto Dominic Rayan, P. Raghavendra Kumar, B. S. Palakshamurthy

**Affiliations:** a Department of Physics, St Philomena’s College (Autonomous), Mysore, Karnataka 570 015, India; b Department of Studies and Research in Chemistry, Tumkur University, Tumkur 572 103, Karnataka, India; c Department of Chemistry, St Philomena’s College (Autonomous), Mysore, Karnataka 570 015, India; d Department of Studies and Research in Physics, U.C.S, Tumkur University, Tumkur, Karnataka 572 103, India

**Keywords:** crystal structure, 1,2,3,4-tetra­hydro­quinoline, C—H⋯O inter­actions, pharmaco­logical activity

## Abstract

The tetra­hydro­pyridine ring of the quinoline system in the title compound, C_14_H_13_ClN_2_O_2_S, adopts a half-chair conformation with the bond-angle sum at the N atom being 350.0°. The dihedral angle between the least-squares planes of the two aromatic rings is 50.13 (11)°. In the crystal, inversion dimers linked by pairs of C—H⋯O hydrogen bonds generate *R*_2_^2^(10) loops. Additional inter­molecular C—H⋯O hydrogen bonds generate *C*(7) chains along [100].

## Abstract 2

In the crystal structure of 1-[(6-chloro­pyridin-3-yl)sulfon­yl]-1,2,3,4-tetra­hydro­quinoline, the tetra­hydro­pyridine ring of the quinoline system adopts a half-chair conformation and the bond-angle sum at the N atom is 350.0°.

## Chemical context   

1,2,3,4-Tetra­hydro­quinoline derivatives play a vital role in developing pharmacological agents and they have been considered as potential drugs (White *et al.*, 1994 ▸; Kokwaro & Taylor, 1990 ▸; Omura & Nakagawa, 1981 ▸) and also antagonists for *N*-methyl-*d*-aspartate (NMDA) receptors at the glycine recognition site (Cai *et al.*, 1996 ▸).
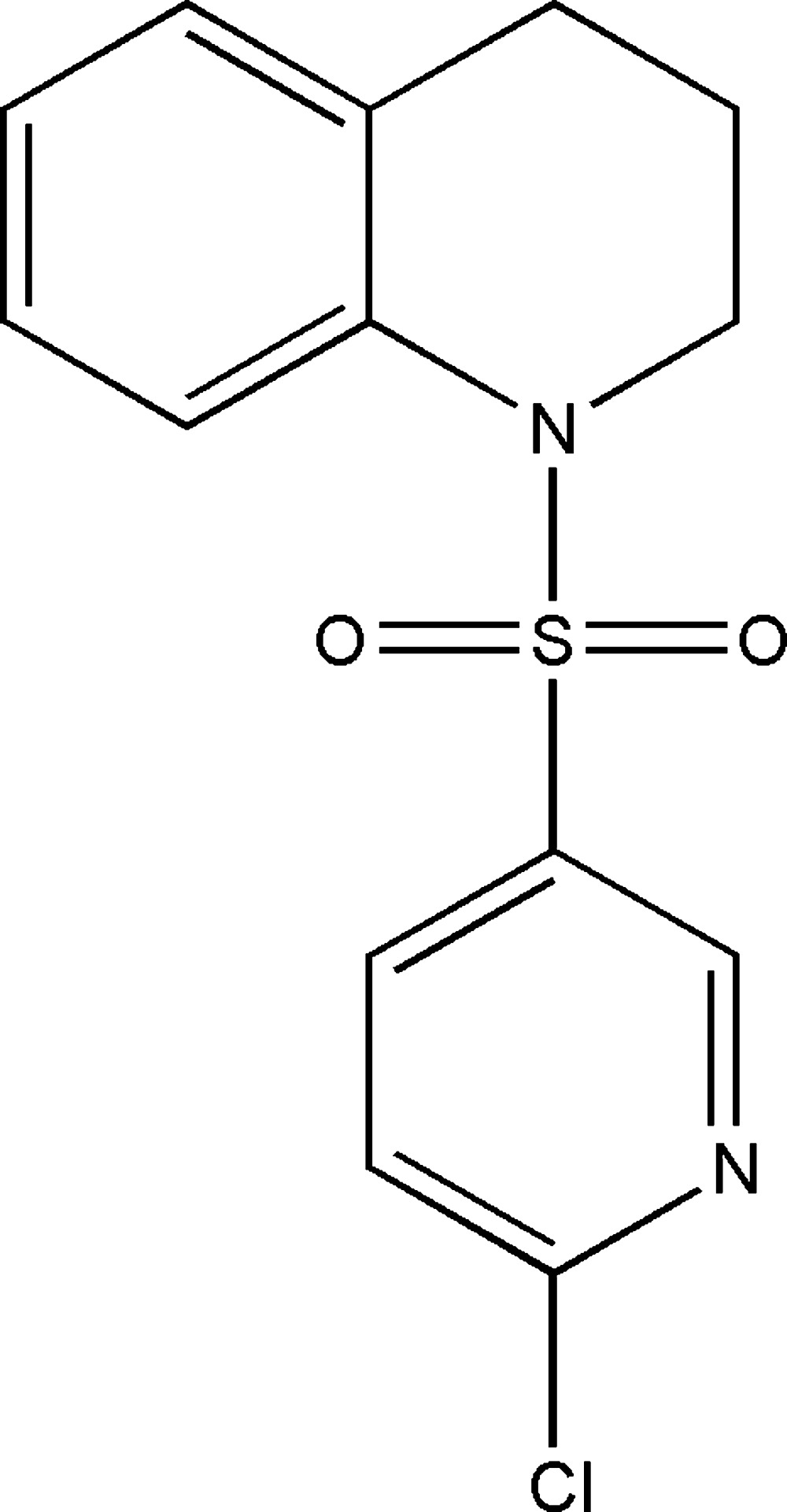


Recently, we have synthesized a series of 1,2,3,4-tetra­hydro­quinoline derivatives and a few mol­ecules in fact exhibit pharmacological activity (unpublished results). In a contin­uation of our work on the derivatives of 1,2,3,4-tetra­hydro­quinolines (Jeyaseelan *et al.*, 2014 ▸, 2015*a* ▸,*b* ▸), we report herein the synthesis and crystal structure of 1-[(6-chloro­pyridin-3-yl)sulfon­yl]-1,2,3,4-tetra­hydro­quinoline, (I).

## Structural commentary   

**Figure 1:**
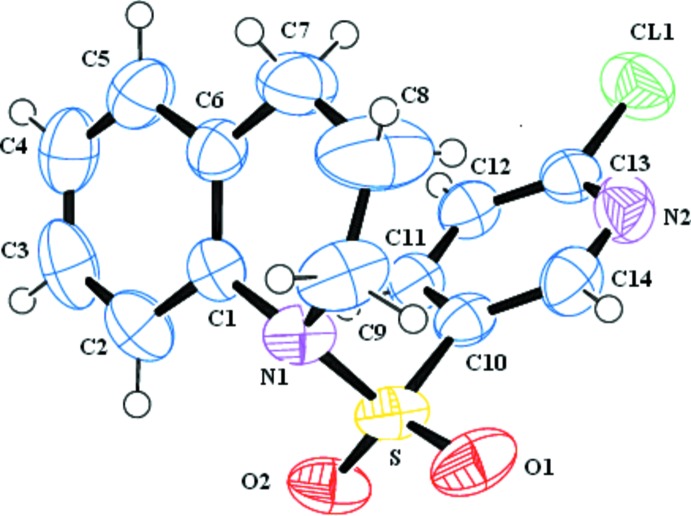
The mol­ecular structure of the title compound, showing displacement ellipsoids drawn at the 50% probability level.

The mol­ecular structure of compound (I) is shown in Fig. 1 ▸. The dihedral angle between the planes of the aromatic rings is 50.13 (11)°. In comparison, the dihedral angle in the 1-tosyl-1,2,3,4-tetra­hydro­quinoline, (II), is 47.74 (9)° (Jeyaseelan *et al.*, 2014 ▸), and in 1-benzyl­sulfonyl-1,2,3,4-tetra­hydro­quinoline, (III), it is 74.15 (10)° (Jeyaseelan *et al.*, 2015*b* ▸). In the structures of compounds (II), (III) and 1-methane­sulfonyl-1,2,3,4-tetra­hydro­quinoline, (IV) (Jeyaseelan *et al.*, 2015*a* ▸), the tetra­hydro­pyridine (C1/C6–C9/N1) ring is in a half-chair conformation, with the methyl­ene C9 atom as the flap. However, the bond-angle sums at the N atom in (I), (II), (III) and (IV) differ somehow, with values of 350.0, 350.2, 354.61 and 347.9°, respectively.

## Supra­molecular features   

**Figure 2:**
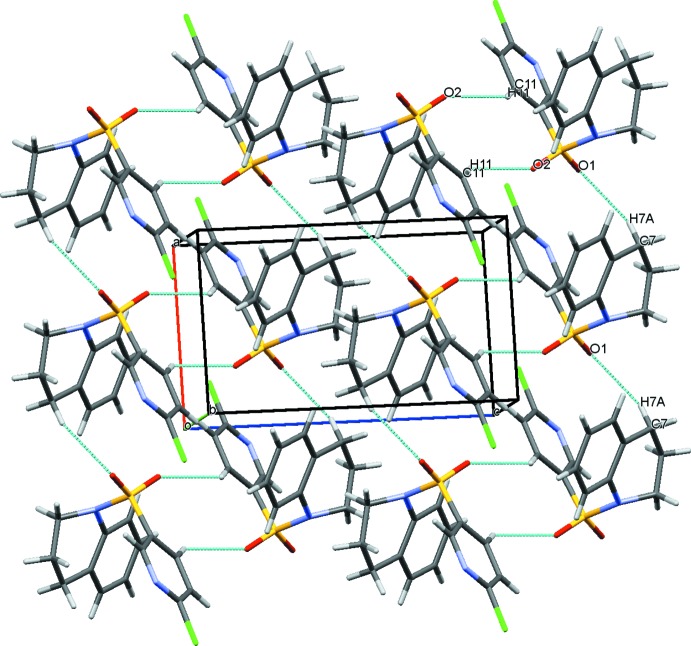
The mol­ecular packing of the title compound. Dashed lines indicate the pairs of C—H⋯O hydrogen bonds which link the mol­ecules into inversion dimers with 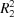
(10) ring motifs and forming *C*(7) chains along [100].

In the crystal, inversion dimers linked by pairs of C11—H11⋯O2 hydrogen bonds generate 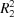
(10) loops. In addition, mol­ecules are linked by C7—H7*A*⋯O1 hydrogen bonds, generating *C*(7) chains along [100], as shown in Fig. 2 ▸. Numerical values of these inter­actions are compiled in Table 1 ▸.

## Synthesis and crystallization   

To an ice-cold solution of 1,2,3,4-tetra­hydro­quinoline (1.332 g, 10 mmol) and tri­ethyl­amine (1.518 g, 15 mmol) in di­chloro­methane (50 ml), a solution of 6-chloro­pyridine-3-sulfonyl chloride (2.332 g, 11 mmol) in di­chloro­methane (20 ml) was added dropwise and stirred for 30 min. The reaction mixture was diluted with di­chloro­methane (150 ml), the organic layer washed with aqueous 5% NaHCO_3_ solution and brine, and dried over anhydrous Na_2_SO_4_. The solvent was evaporated under reduced pressure to give 1-[(6-chloro­pyridin-3-yl)sulfon­yl]-1,2,3,4-tetra­hydro­quinoline, (I). The product was recrystallized from a mixture of di­chloro­methane and *n*-hexane (1:1 *v*/*v*) to obtain crystals suitable for X-ray diffraction studies.

## Refinement details   

Crystal data, data collection and structure refinement details are summarized in Table 2 ▸. H atoms were positioned with idealized geometry using a riding-model approximation, with C—H = 0.93 Å and *U*_iso_(H) = 1.2*U*_eq_(C) for aromatic H atoms and with C—H = 0.97 Å and *U*_iso_(H) = 1.2*U*_eq_(C) for methyl­ene H atoms.

## Supplementary Material

Crystal structure: contains datablock(s) I, New_Global_Publ_Block. DOI: 10.1107/S2056989015008099/wm5147sup1.cif

Structure factors: contains datablock(s) I. DOI: 10.1107/S2056989015008099/wm5147Isup2.hkl

Supporting information file. DOI: 10.1107/S2056989015008099/wm5147Isup3.cml

CCDC reference: 1061311

Additional supporting information:  crystallographic information; 3D view; checkCIF report

## Figures and Tables

**Table 1:** Hydrogen-bond geometry (, )

*D*H*A*	*D*H	H*A*	*D* *A*	*D*H*A*
C11H11O2^i^	0.93	2.60	3.309(3)	134
C7H7*A*O1^ii^	0.97	2.66	3.586(5)	160

Symmetry codes: (i) 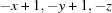; (ii) 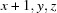.

**Table 2:** Experimental details

Crystal data	
Chemical formula	C_14_H_13_ClN_2_O_2_S
*M*_r_	308.77
Crystal system, space group	Triclinic, *P* 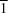
Temperature (K)	296
*a*, *b*, *c* ()	6.5661(10), 10.2595(18), 11.3490(19)
, , ()	69.101(7), 88.219(7), 77.238(7)
*V* (^3^)	695.6(2)
*Z*	2
Radiation type	Mo *K*
(mm^1^)	0.43
Crystal size (mm)	0.23 0.18 0.16

Data collection	
Diffractometer	Bruker APEXII CCD
Absorption correction	Multi-scan (*SADABS*; Bruker, 2013 ▸)
*T*_min_, *T*_max_	0.912, 0.934
No. of measured, independent and observed [*I* > 2(*I*)] reflections	9865, 2454, 1980
*R*_int_	0.053
(sin /)_max_ (^1^)	0.595

Refinement	
*R*[*F* ^2^ > 2(*F* ^2^)], *wR*(*F* ^2^), *S*	0.050, 0.146, 1.09
No. of reflections	2454
No. of parameters	181
H-atom treatment	H-atom parameters constrained
_max_, _min_ (e ^3^)	0.59, 0.43

Computer programs: *APEX2* and *SAINT* (Bruker, 2013 ▸), *SHELXS97* (Sheldrick, 2008 ▸), *SHELXL2014* (Sheldrick, 2015 ▸), *ORTEP-3 for Windows* (Farrugia, 2012 ▸) and *Mercury* (Macrae *et al.*, 2008 ▸).

## supplementary crystallographic information

### Crystal data

**Table:**

C_14_H_13_ClN_2_O_2_S	*F*(000) = 320
*M**_r_* = 308.77	prism
Triclinic, *P*1	*D*_x_ = 1.474 Mg m^−^^3^
Hall symbol: -P 1	Melting point: 413 K
*a* = 6.5661 (10) Å	Mo *K*α radiation, λ = 0.71073 Å
*b* = 10.2595 (18) Å	Cell parameters from 1980 reflections
*c* = 11.3490 (19) Å	θ = 1.9–25.0°
α = 69.101 (7)°	µ = 0.43 mm^−^^1^
β = 88.219 (7)°	*T* = 296 K
γ = 77.238 (7)°	Prism, colourless
*V* = 695.6 (2) Å^3^	0.23 × 0.18 × 0.16 mm
*Z* = 2	

### Data collection

**Table:**

Bruker APEXII CCD diffractometer	2454 independent reflections
Radiation source: fine-focus sealed tube	1980 reflections with *I* > 2σ(*I*)
Graphite monochromator	*R*_int_ = 0.053
Detector resolution: 2.01 pixels mm^-1^	θ_max_ = 25.0°, θ_min_ = 1.9°
phi and ω scans	*h* = −7→7
Absorption correction: multi-scan (*SADABS*; Bruker, 2013)	*k* = −12→12
*T*_min_ = 0.912, *T*_max_ = 0.934	*l* = −13→13
9865 measured reflections	

### Refinement

**Table:**

Refinement on *F*^2^	Primary atom site location: structure-invariant direct methods
Least-squares matrix: full	Secondary atom site location: difference Fourier map
*R*[*F*^2^ > 2σ(*F*^2^)] = 0.050	Hydrogen site location: inferred from neighbouring sites
*wR*(*F*^2^) = 0.146	H-atom parameters constrained
*S* = 1.09	*w* = 1/[σ^2^(*F*_o_^2^) + (0.0677*P*)^2^ + 0.3614*P*] where *P* = (*F*_o_^2^ + 2*F*_c_^2^)/3
2454 reflections	(Δ/σ)_max_ < 0.001
181 parameters	Δρ_max_ = 0.59 e Å^−^^3^
0 restraints	Δρ_min_ = −0.43 e Å^−^^3^
0 constraints	

### Special details

**Table:**

Geometry. All e.s.d.'s (except the e.s.d. in the dihedral angle between two l.s. planes) are estimated using the full covariance matrix. The cell e.s.d.'s are taken into account individually in the estimation of e.s.d.'s in distances, angles and torsion angles; correlations between e.s.d.'s in cell parameters are only used when they are defined by crystal symmetry. An approximate (isotropic) treatment of cell e.s.d.'s is used for estimating e.s.d.'s involving l.s. planes.

### Fractional atomic coordinates and isotropic or equivalent isotropic displacement parameters (Å^2^)

**Table:**

	*x*	*y*	*z*	*U*_iso_*/*U*_eq_	
O1	0.3014 (3)	0.1696 (3)	0.3092 (2)	0.0753 (7)	
S	0.40371 (11)	0.28345 (8)	0.24768 (6)	0.0562 (3)	
Cl1	1.24374 (14)	0.01308 (10)	0.08760 (9)	0.0853 (3)	
C10	0.6392 (4)	0.2111 (3)	0.1933 (2)	0.0479 (6)	
N1	0.4673 (3)	0.3394 (2)	0.35739 (19)	0.0532 (6)	
O2	0.3005 (3)	0.4047 (2)	0.14401 (19)	0.0712 (6)	
C11	0.7513 (4)	0.2997 (3)	0.1091 (2)	0.0504 (6)	
H11	0.6991	0.3983	0.0764	0.060*	
N2	0.9018 (4)	0.0058 (3)	0.2047 (2)	0.0657 (7)	
C1	0.5901 (4)	0.4468 (3)	0.3217 (2)	0.0479 (6)	
C13	1.0055 (4)	0.0935 (3)	0.1264 (2)	0.0545 (7)	
C6	0.7874 (4)	0.4136 (3)	0.3794 (3)	0.0542 (7)	
C12	0.9387 (4)	0.2403 (3)	0.0750 (3)	0.0545 (7)	
H12	1.0188	0.2965	0.0190	0.065*	
C14	0.7193 (5)	0.0656 (3)	0.2371 (3)	0.0615 (8)	
H14	0.6417	0.0064	0.2921	0.074*	
C2	0.5110 (5)	0.5812 (3)	0.2320 (3)	0.0678 (8)	
H2	0.3760	0.6042	0.1966	0.081*	
C5	0.9038 (5)	0.5175 (4)	0.3419 (3)	0.0690 (8)	
H5	1.0362	0.4975	0.3798	0.083*	
C9	0.5138 (5)	0.2322 (4)	0.4875 (3)	0.0733 (10)	
H9A	0.4544	0.2766	0.5470	0.088*	
H9B	0.4466	0.1542	0.4963	0.088*	
C3	0.6339 (7)	0.6804 (3)	0.1957 (3)	0.0812 (10)	
H3	0.5830	0.7694	0.1335	0.097*	
C4	0.8292 (6)	0.6494 (4)	0.2502 (3)	0.0766 (10)	
H4	0.9112	0.7169	0.2255	0.092*	
C7	0.8693 (6)	0.2732 (4)	0.4825 (4)	0.0796 (10)	
H7A	0.9980	0.2263	0.4563	0.096*	
H7B	0.9042	0.2917	0.5566	0.096*	
C8	0.7326 (7)	0.1753 (5)	0.5187 (4)	0.124 (2)	
H8A	0.7792	0.1037	0.4804	0.149*	
H8B	0.7507	0.1261	0.6095	0.149*	

### Atomic displacement parameters (Å^2^)

**Table:**

	*U*^11^	*U*^22^	*U*^33^	*U*^12^	*U*^13^	*U*^23^
O1	0.0680 (14)	0.0963 (16)	0.0687 (13)	−0.0494 (13)	0.0085 (11)	−0.0202 (12)
S	0.0469 (4)	0.0698 (5)	0.0465 (4)	−0.0217 (3)	−0.0027 (3)	−0.0090 (3)
Cl1	0.0736 (6)	0.0948 (7)	0.0859 (6)	0.0056 (5)	−0.0027 (5)	−0.0446 (5)
C10	0.0520 (15)	0.0511 (15)	0.0373 (12)	−0.0186 (12)	−0.0063 (11)	−0.0069 (11)
N1	0.0489 (12)	0.0633 (14)	0.0406 (11)	−0.0183 (11)	0.0032 (9)	−0.0072 (10)
O2	0.0547 (12)	0.0850 (15)	0.0553 (11)	−0.0067 (11)	−0.0148 (9)	−0.0068 (11)
C11	0.0571 (16)	0.0441 (14)	0.0429 (13)	−0.0133 (12)	−0.0019 (12)	−0.0056 (11)
N2	0.0813 (19)	0.0509 (14)	0.0591 (15)	−0.0116 (13)	−0.0060 (13)	−0.0140 (12)
C1	0.0511 (15)	0.0463 (14)	0.0435 (13)	−0.0087 (12)	0.0095 (11)	−0.0146 (11)
C13	0.0566 (16)	0.0595 (17)	0.0472 (14)	−0.0088 (13)	−0.0096 (12)	−0.0203 (13)
C6	0.0566 (16)	0.0517 (16)	0.0551 (15)	−0.0155 (13)	0.0029 (13)	−0.0184 (13)
C12	0.0561 (16)	0.0596 (17)	0.0482 (14)	−0.0218 (14)	0.0053 (12)	−0.0149 (13)
C14	0.078 (2)	0.0509 (17)	0.0508 (15)	−0.0255 (16)	0.0011 (14)	−0.0056 (13)
C2	0.0673 (19)	0.0558 (18)	0.0628 (18)	−0.0001 (15)	0.0048 (15)	−0.0088 (14)
C5	0.070 (2)	0.070 (2)	0.077 (2)	−0.0312 (17)	0.0105 (16)	−0.0290 (17)
C9	0.079 (2)	0.093 (2)	0.0399 (15)	−0.0443 (19)	0.0005 (14)	−0.0004 (15)
C3	0.105 (3)	0.0411 (16)	0.081 (2)	−0.0057 (18)	0.025 (2)	−0.0101 (15)
C4	0.098 (3)	0.060 (2)	0.084 (2)	−0.0356 (19)	0.033 (2)	−0.0315 (18)
C7	0.066 (2)	0.068 (2)	0.086 (2)	−0.0170 (17)	−0.0195 (17)	−0.0029 (17)
C8	0.110 (3)	0.118 (3)	0.089 (3)	−0.050 (3)	−0.042 (3)	0.047 (3)

### Geometric parameters (Å, º)

**Table:**

O1—S	1.428 (2)	C6—C5	1.385 (4)
S—O2	1.423 (2)	C6—C7	1.492 (4)
S—O1	1.428 (2)	C12—H12	0.9300
S—N1	1.644 (2)	C14—H14	0.9300
S—C10	1.756 (3)	C2—C3	1.378 (5)
Cl1—C13	1.723 (3)	C2—H2	0.9300
C10—C14	1.376 (4)	C5—C4	1.374 (5)
C10—C11	1.383 (3)	C5—H5	0.9300
N1—C1	1.443 (3)	C9—C8	1.430 (5)
N1—C9	1.484 (3)	C9—H9A	0.9700
C11—C12	1.358 (4)	C9—H9B	0.9700
C11—H11	0.9300	C3—C4	1.362 (5)
N2—C13	1.314 (4)	C3—H3	0.9300
N2—C14	1.325 (4)	C4—H4	0.9300
C1—C2	1.386 (4)	C7—C8	1.437 (5)
C1—C6	1.387 (4)	C7—H7A	0.9700
C13—C12	1.378 (4)	C8—H8A	0.9700
			
O2—S—O1	120.12 (13)	C3—C2—C1	119.5 (3)
O2—S—N1	108.30 (13)	C3—C2—H2	120.2
O1—S—N1	106.51 (12)	C1—C2—H2	120.2
O2—S—C10	106.62 (12)	C4—C5—C6	121.9 (3)
O1—S—C10	107.97 (14)	C4—C5—H5	119.1
N1—S—C10	106.63 (12)	C6—C5—H5	119.1
C14—C10—C11	118.8 (3)	C8—C9—N1	113.3 (3)
C14—C10—S	120.6 (2)	C8—C9—H9A	108.9
C11—C10—S	120.6 (2)	N1—C9—H9A	108.9
C1—N1—C9	115.2 (2)	C8—C9—H9B	108.9
C1—N1—S	117.64 (16)	N1—C9—H9B	108.9
C9—N1—S	117.2 (2)	H9A—C9—H9B	107.7
C12—C11—C10	118.9 (3)	C4—C3—C2	120.7 (3)
C12—C11—H11	120.6	C4—C3—H3	119.6
C10—C11—H11	120.6	C2—C3—H3	119.6
C13—N2—C14	116.3 (2)	C3—C4—C5	119.4 (3)
C2—C1—C6	120.7 (3)	C3—C4—H4	120.3
C2—C1—N1	120.4 (3)	C5—C4—H4	120.3
C6—C1—N1	118.8 (2)	C8—C7—C6	116.5 (3)
N2—C13—C12	125.4 (3)	C8—C7—H7A	108.2
N2—C13—Cl1	115.3 (2)	C6—C7—H7A	108.2
C12—C13—Cl1	119.2 (2)	C8—C7—H7B	108.2
C5—C6—C1	117.8 (3)	C6—C7—H7B	108.2
C5—C6—C7	120.7 (3)	H7A—C7—H7B	107.3
C1—C6—C7	121.5 (2)	C9—C8—C7	118.0 (4)
C11—C12—C13	117.4 (3)	C9—C8—H8A	107.8
C11—C12—H12	121.3	C7—C8—H8A	107.8
C13—C12—H12	121.3	C9—C8—H8B	107.8
N2—C14—C10	123.1 (3)	C7—C8—H8B	107.8
N2—C14—H14	118.4	H8A—C8—H8B	107.1
C10—C14—H14	118.4		
			
O2—S—C10—C14	−145.4 (2)	C9—N1—C1—C6	27.0 (4)
O2—S—C10—C14	−145.4 (2)	S—N1—C1—C6	−117.9 (2)
O1—S—C10—C14	−15.0 (3)	C14—N2—C13—C12	−1.0 (4)
O1—S—C10—C14	−15.0 (3)	C14—N2—C13—Cl1	179.2 (2)
O1—S—C10—C14	−15.0 (3)	C2—C1—C6—C5	−1.8 (4)
N1—S—C10—C14	99.1 (2)	N1—C1—C6—C5	178.8 (2)
O2—S—C10—C11	37.0 (2)	C2—C1—C6—C7	175.7 (3)
O2—S—C10—C11	37.0 (2)	N1—C1—C6—C7	−3.7 (4)
O1—S—C10—C11	167.3 (2)	C10—C11—C12—C13	0.4 (4)
O1—S—C10—C11	167.3 (2)	N2—C13—C12—C11	0.9 (4)
O1—S—C10—C11	167.3 (2)	Cl1—C13—C12—C11	−179.2 (2)
N1—S—C10—C11	−78.6 (2)	C13—N2—C14—C10	−0.3 (4)
O2—S—N1—C1	−54.9 (2)	C11—C10—C14—N2	1.5 (4)
O2—S—N1—C1	−54.9 (2)	S—C10—C14—N2	−176.1 (2)
O1—S—N1—C1	174.7 (2)	C6—C1—C2—C3	3.1 (4)
O1—S—N1—C1	174.7 (2)	N1—C1—C2—C3	−177.6 (3)
O1—S—N1—C1	174.7 (2)	C1—C6—C5—C4	−0.3 (5)
C10—S—N1—C1	59.5 (2)	C7—C6—C5—C4	−177.8 (3)
O2—S—N1—C9	161.0 (2)	C1—N1—C9—C8	−46.6 (5)
O2—S—N1—C9	161.0 (2)	S—N1—C9—C8	98.4 (4)
O1—S—N1—C9	30.5 (2)	C1—C2—C3—C4	−2.3 (5)
O1—S—N1—C9	30.5 (2)	C2—C3—C4—C5	0.2 (5)
O1—S—N1—C9	30.5 (2)	C6—C5—C4—C3	1.1 (5)
C10—S—N1—C9	−84.6 (2)	C5—C6—C7—C8	177.1 (4)
C14—C10—C11—C12	−1.6 (4)	C1—C6—C7—C8	−0.3 (6)
S—C10—C11—C12	176.12 (19)	N1—C9—C8—C7	43.5 (6)
C9—N1—C1—C2	−152.4 (3)	C6—C7—C8—C9	−20.5 (7)
S—N1—C1—C2	62.8 (3)		

### Hydrogen-bond geometry (Å, º)

**Table:**

*D*—H···*A*	*D*—H	H···*A*	*D*···*A*	*D*—H···*A*
C11—H11···O2^i^	0.93	2.60	3.309 (3)	134
C7—H7*A*···O1^ii^	0.97	2.66	3.586 (5)	160

Symmetry codes: (i) −*x*+1, −*y*+1, −*z*; (ii) *x*+1, *y*, *z*.
